# Intricate crosstalk between MYB and noncoding RNAs in cancer

**DOI:** 10.1186/s12935-021-02362-4

**Published:** 2021-12-07

**Authors:** Dingyu Hu, Wenjun Shao, Li Liu, Yanyan Wang, Shunling Yuan, Zhaoping Liu, Jing Liu, Ji Zhang

**Affiliations:** 1 The First Affiliated Hospital, Department of Rheumatology, Hengyang Medical School, University of South China, Hengyang, 421001 Hunan China; 2 Hunan Province Key Laboratory of Basic and Applied Hematology, Molecular Biology Research Center & Center for Medical Genetics, School of Life Sciences, Central South University, Changsha, 410078 Hunan China; 3 Department of Clinical Laboratory, Shenzhen Traditional Chinese Medicine Hospital, Shenzhen, 518033 Guangdong China

**Keywords:** MYB, Noncoding RNAs, LncRNAs, MiRNAs, Tumorigenesis

## Abstract

MYB is often overexpressed in malignant tumors and plays a carcinogenic role in the initiation and development of cancer. Deletion of the MYB regulatory C-terminal domain may be a driving mutation leading to tumorigenesis, therefore, different tumor mechanisms produce similar MYB proteins. As MYB is a transcription factor, priority has been given to identifying the genes that it regulates. All previous attention has been focused on protein-coding genes. However, an increasing number of studies have suggested that MYB can affect the complexity of cancer progression by regulating tumor-associated noncoding RNAs (ncRNAs), such as microRNAs, long-non-coding RNAs and circular RNAs. ncRNAs can regulate the expression of numerous downstream genes at the transcription, RNA processing and translation levels, thereby having various biological functions. Additionally, ncRNAs play important roles in regulating MYB expression. This review focuses on the intricate crosstalk between oncogenic MYB and ncRNAs, which play a pivotal role in tumorigenesis, including proliferation, apoptosis, angiogenesis, metastasis, senescence and drug resistance. In addition, we discuss therapeutic strategies for crosstalk between MYB and ncRNAs to prevent the occurrence and development of cancer.

## Introduction

**Fig. 1:**
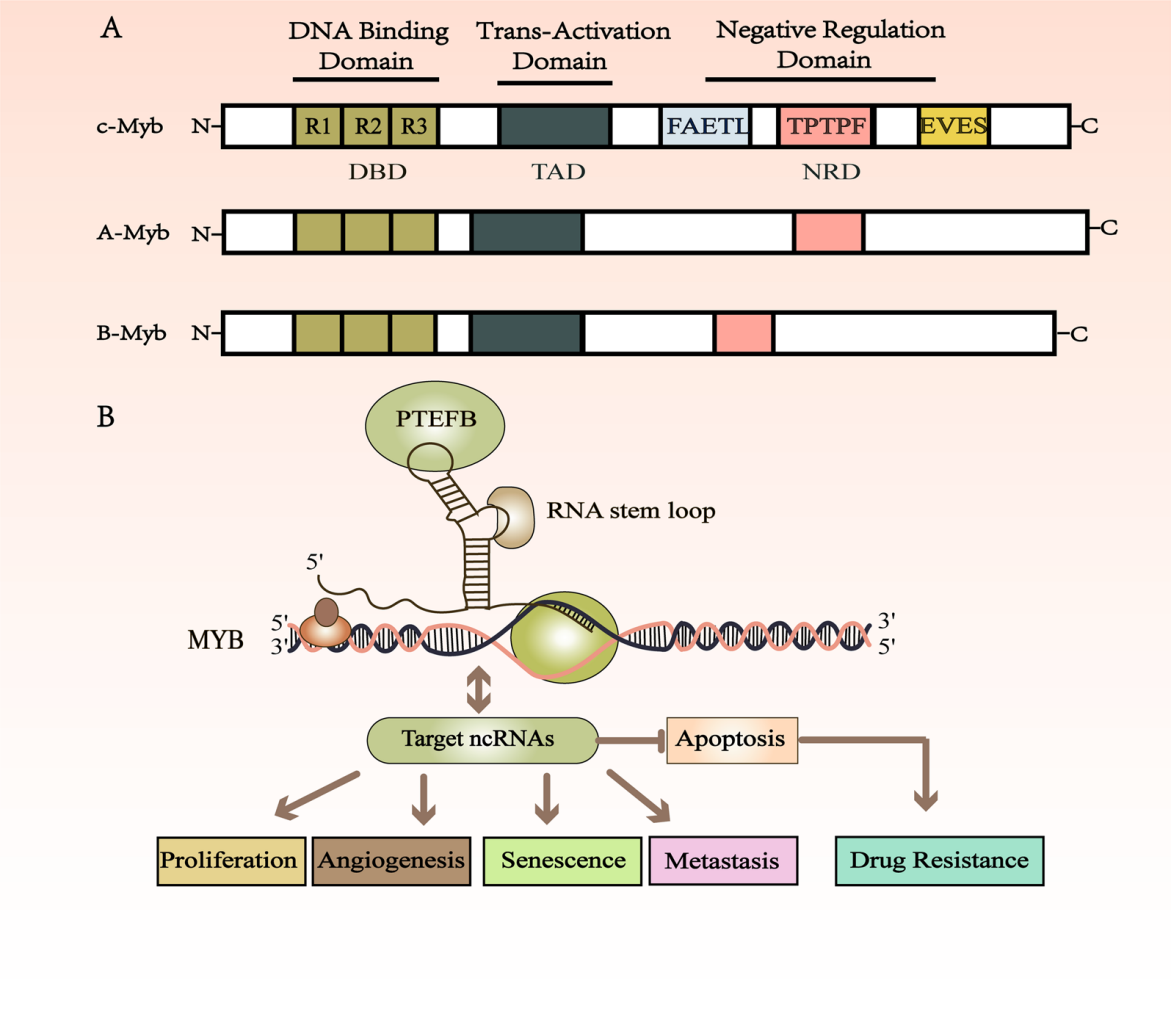
The diagram shows the schematic structure of the gene and protein of A-MYB, B-MYB and c-MYB. **A** The domain structures of A-MYB, B-MYB, and c-MYB. The MYB protein is diagrammed, with N-terminal on the left and C-terminal on the right. The labels at the bottom of the diagram indicate conserved domains. The *MYB* gene is located on chromosome 6q23.3 and encodes a transcription factor with an N-terminal DNA binding domain (DBD), a central transactivation domain (TAD), and a negative regulatory domain (NRD). Oncogenic activity requires the FAETL domain, the TPTPF domain conserved in the other MYB proteins, and the EVES domain that is involved in intra-molecular interactions and negative regulation. **B** MYB transcriptional elongation regulation model, and the effect of the interaction between ncRNAs and MYB on the occurrence and development of tumor cells

The MYB gene was discovered from *virus MYB (V-MYB)*, which is the oncogene of avian myeloblastosis virus (AMV) and E26 (another avian virus), and is considered a causative the oncogene of avian myeloma and lymphoma in birds. This has led, to the hypothesis that aberrant activation of vertebrate MYB could also cause cancer [1]. Moreover, the nucleotide sequence of the promoter region of the MYB proto-oncogene was detected in mice, humans, lizards, frogs, and carp, indicating that this evolutionarily conserved element is involved in the regulation of MYB proto-oncogene expression in vertebrates [2]. The MYB protein contains a DNA-binding domain (DBD) at the N-terminal, which consists of three tandem repeat domains of approximately 50 amino acids containing tryptophan, named R1, R2 and R3; a conserved C-terminal negative regulatory domain (NRD); and a transactivation domain (TAD) located in the central part of the protein (Fig. 1) [3]. Evidence suggests that deletion of MYB regulatory C-terminal domain may be a driving mutation leading to tumorigenesis [1]. In leukemia samples, enhanced alternative RNA splicing produces mutated MYB gene transcripts [4]. Moreover, recurrent t (6;9) (q22-23; p23-24) translocation in adenoid cystic carcinoma fuse MYB gene on chromosome 6 to NFIB gene on chromosome 9 [5]. Cellular MYB (c-MYB) is a homolog of *v-MYB*, which paved the way for the discovery of two closely related family members, MYBL1 (A-MYB) and MYBL2 (B-MYB) [6, 7]. Although their structures are similar, they may have unique biological functions. Different MYB proteins interact with distinct cofactors, and their expression is usually nonoverlapping [8–10]. A-MYB expression is limited to developing mammary glands, spermatogenic tissues, central nervous system, and T and B cells [11]. B-MYB seems to be ubiquitously expressed in normal tissues and is overexpressed in many cancers, especially leukemia, colorectal cancer, esophageal squamous cancer, bladder carcinoma and breast cancer [12–16]. The conditional inactivation of B-MYB in vivo will lead to depletion of hematopoietic stem cell (HSC) bank and a massive reduction in mature lymphocytes, erythrocytes and myelocytes [17]. C-MYB encodes a transcriptional activator that is critical for the development of the hematopoietic system [18]. A study of MYB knockout mice showed that the precise expression of MYB gene had differential effects on the development of T and B cells, bone marrow production, erythropoiesis and HSC self-renewal [7]. Numerous studies have shown that MYB overexpression can promote the growth of tumor cells [19–21]. Previous studies have shown that MYB inhibition can impair the growth, migration and invasion of cancer cells, suggesting that inhibition of MYB may be a potential cancer treatment strategy [19, 22, 23].

MYB acts as a transcriptional activator by binding to a specific sequence, called MYB binding site (MBS) [24]. Interestingly, *MYB* encodes one or more proteins that can interact with other transcription factors such as ETS-2, NFM, and CEBP [25]. Evidence suggests that there are systematic changes in the processing of RNA in cancer. These changes can be observed in the form of noncoding RNAs (ncRNAs) [26]. In the past decade, booming bioinformatics and deep sequencing technology have enabled the identification and annotation of tens of thousands of ncRNAs [27, 28]. These ncRNAs mainly include long-non-coding RNAs (lncRNAs), microRNAs (miRNAs), and cyclic RNAs (circRNAs) [29].

Over the past few years, these ncRNAs have proven to have a wide range of potential for controlling gene expression [30, 31]. LncRNAs are transcripts with a length of more than 200 nucleotides and are rapidly becoming a new type of transcript related to a variety of cellular and biological processes. The role of lncRNAs in cancer is mainly reflected in two aspects, as RNA molecules and by encoding peptides or proteins [32]. Furthermore, their abnormal expression and mutation are closely related to tumorigenesis, metastasis and tumor stage in leukemia, prostate cancer, and breast cancer [33–35]. Furthermore, miRNAs disturb expression of genes or degrade messenger RNA (mRNA) translation by binding to complementary target genes [36]. MiRNAs are aberrantly expressed in a variety of tumors. The first example is miR-15a and miR-16, which provide further clues to their role in the pathogenesis of B-lymphocytic leukemia (B-CLL) [37]. CircRNAs are a class of single-stranded RNAs with closed circular structures that play significant roles in the initiation and progression of cancer [38]. Interestingly, Lee et al. found that the transcription factor (TF) c-MYB participates in the regulation of 48 miRNAs [39]. In 2009, Zhao et al. found that the c-MYB-miR-15a autoregulation feedback loop plays an important role in human hematopoiesis and confirmed that MYB plays a regulatory role in ncRNA expression [40]. In this review, we focus on the complex crosstalk between ncRNAs and MYB in the pathogenesis and development of cancers.

### MYB interacts with miRNAs

**Fig. 2:**
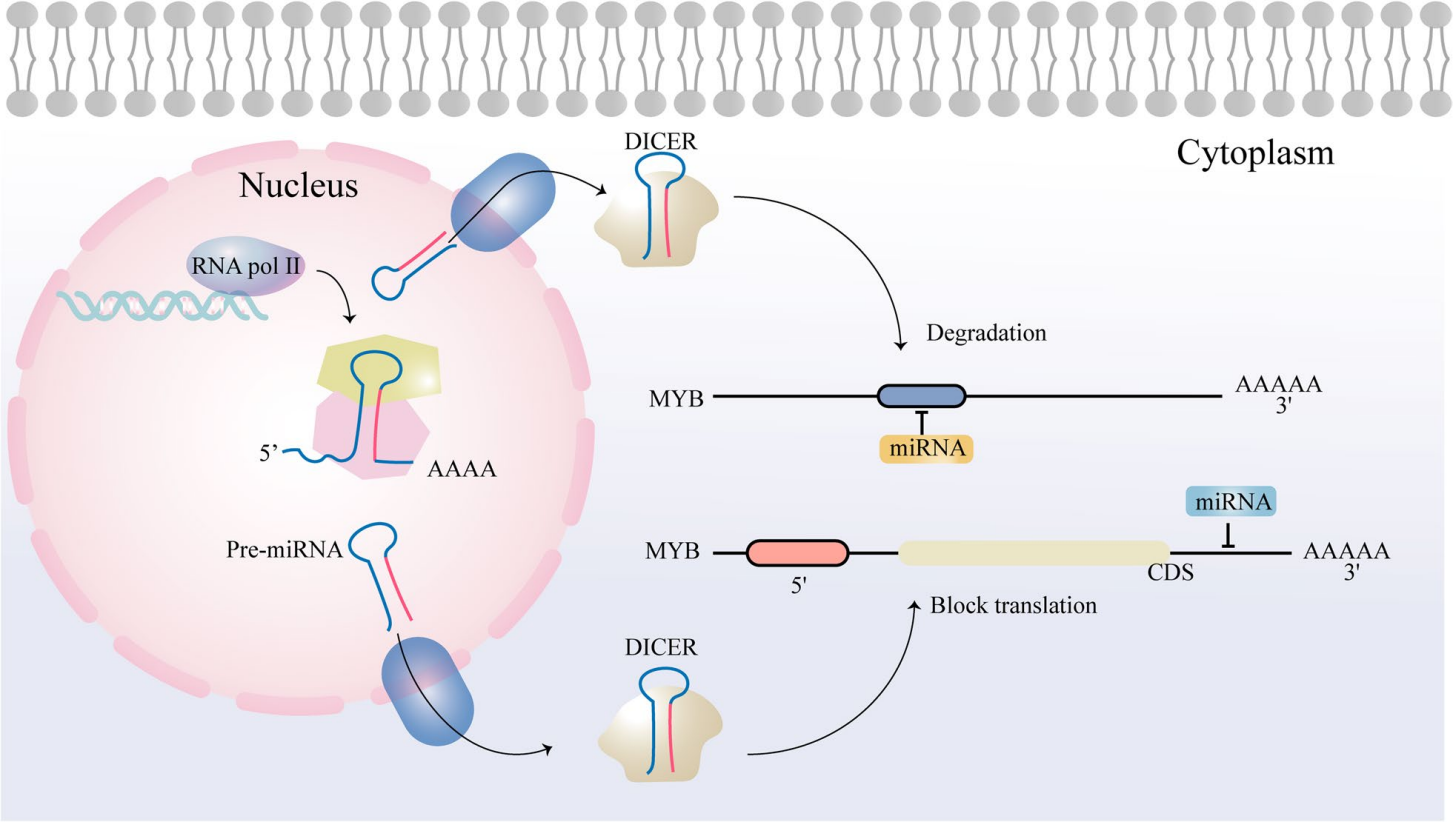
Schematic of the biogenesis of miRNA and the functional mechanism of miRNA. MiRNAs directly bind to the 3′UTR of *MYB* to regulate *MYB* expression. If miRNA and MYB are completely complementary, then the combination of these miRNAs causes the degradation of MYB

In recent years, the relationship between MYB and miRNAs has been studied extensively [41]. MYB can be used as a transcriptional activator to induce up-regulation of miRNA expression. Conversely, miRNAs play a crucial role in transcriptional regulation of *MYB* gene expression by binding to complementary sequences in its 3′-untranslated region (UTR) (Fig. 2).

### Regulation of miRNAs by MYB

Many studies have shown that MYB participates in the regulation of miRNA expression and is involved in the pathogenesis and development of various forms of cancer (Table 1). For example, *MYB* binds to MBS-C in the miR-143 promoter, thereby transactivating miR-143 to affect the proliferation and differentiation of nasopharyngeal carcinoma cells [42]. *MYB* physically binds to the promoter of miR-155HG and activates its transcription in chronic B-CLL [43]. The proapoptotic effects of miR-148a have been demonstrated in previous study [44]. MYB can transactivate BCL-2 by identifying transcription factor binding sites (TFBs) and indirectly regulate BCL-2 by inhibiting miR-148a [45, 46].

**Table 1:** The role of miRNAs targeted by MYB in cancer development

MYB-targeted miRNAs	Expression	Targeting	Cellular processes	Tumor types	In vitro model	In vivo model	Refs.
miR-520-h	Upregulated	Smad7, MAGI1	Metastasis	RCC, EOC	RCC cell lines (786-O, A-498, OS-RC-2, ACHN, CAKI-1, SKRC39 and HK-2)	BALB/C nude mice	[48]
miR-130a	Upregulated	NDRG2	Cell proliferation, metastasis	SACC	SACC-83, SACCLM cells	Female BALB/C-nu/nu nude mice	[47]
miR-155	Upregulated	PU.1	Cell proliferation, metastasis, cell cycle	AML		PU.1/p53 double-mutant mice	[51]
miR-17-92	Upregulated	FRZB, p21, E2F1	Senescence	Ph-positive leukemia	BV173, SUP-B15 and K562	NOD/SCID gamma mice	[131]
miR-143	Upregulated	Ras	Cell proliferation, apoptosis, DNA repair, metastasis.	Nasopharyngeal carcinoma	Human NPC cell lines, c666-1, 5-8F, CNE1 and CNE2		[42]
miR-1258	Downregulated	SP-1, GRB2	Metastasis, cell cycle, senescence	OSCC, NSCLC	OSCC cell lines (SCC-9, SCC-15); Human NSCLC cell lines (A549, SPCA1, H1299, H358, PC9, 95D,16HBE); HUVEC and HEK293 cells	NOD/SCID mice	[49, 129]
miR-148a	Downregulated	BCL2	Apoptosis	Colorectal cancer	RKO, LOVO, W480 cells		[111]

AML, acute myeloid leukemia; EOC, epithelial ovarian cancer; HUVEC, human umbilical vein endothelial cell; NSCLC, non-small cell lung cancer; NPC, nasopharyngeal carcinoma; OSCC, oral squamous cell carcinoma; RCC, renal cell carcinoma; SACC, salivary adenoid cystic carcinoma

Notably, many MYB-regulated miRNAs affect the development of cancer by targeting certain transcription factors and tumor suppressors [47, 48]. MiR-1258 is a key target gene of MYB, and its oncogenic effect is achieved by targeting transcription factor SP-1. There is evidence that upregulated SP-1 plays a crucial role in cell proliferation and metastasis of various tumors; thus, it is considered to be a negative factor in cancer prognosis [49, 50]. In tumor cells with high *MYB* expression, *MYB* induces miR-130a expression, which inhibits the expression of tumor suppressor NDRG2 by targeting its 3'-UTR [47]. Studies have shown that *MYB* promotes the transcriptional activity of miR-520-h, and upregulated miR-520-h can directly downregulate membrane-associated guanylate kinase and reverse repeat member 1 (MAGI1) expression [48]. Moreover, in acute myeloid leukemia (AML), *MYB* expression activates miR-155 and inhibits transcription factor PU.1. The highly activated MYB/miR-155/PU.1 pathway may be involved in the pathogenesis and invasiveness of AML [51]. Interestingly, transcriptional regulators exert an enormous effect in modulating the expression of miRNA by targeting MYB. Y-box binding protein 1 (YB-1), a DNA and RNA-binding protein family member, is a multifunctional oncoprotein that plays a critical role in cell processes [52, 53]. In laryngeal squamous cell carcinoma (LSCC), YB-1 induces miR-155 expression through *MYB* and promotes cancer development [54].

In addition to the above unidirectional regulation of MYB and miRNA, there is also a feedback loop between MYB and miRNA. MYB transcription factors directly bind to the upstream promoter region of miR-15a, and the expression of miR-15a is caused by this binding [40]. Conversely, miR-15a can repress *MYB* expression. The destruction of this feedback loop may lead to abnormal MYB activity and malignant transformation [40]. The role of the miR-200 family in the treatment of breast cancer has been demonstrated [55–57]. In recent years, some studies have shown that miR-200 regulates the development of breast cancer by directly negatively regulating the expression of *MYB* [58]. Interestingly, *MYB* positively controls the expression of miR-200, but this expression depends on potent repressors and miR-200 promoter methylation [59].

### MiRNAs affecting MYB expression

Dozens of miRNAs have been described to regulate MYB expression by inhibiting its translation or degradation of its mRNA (Table 2). For example, both miR-143-3p and miR-29 negatively regulate the expression of *MYB* by directly binding to the 3′UTR of *MYB* [60, 61]. Many miRNAs show reduced levels in cancer, the inhibition of *MYB* by these miRNAs is removed, and the expression of *MYB* is upregulated. Therefore, the ordinarily high MYB level may be due to the decreased expression of tumor suppressor miRNAs, such as miR-96, miR-34a, miR-15a/16, miR-193b-3p, miR-548c-3p and miR-155 [62–69].

**Table 2:** The role of miRNAs targeting MYB in a variety of cancers

Targeting MYB	Cellular processes	Tumor types	In vitro model	In vivo model	Refs.
miR-200	Cell proliferation, resistance	Breast cancer	MCF-7, T47D cells		[58]
miR-143-3p	Cell proliferation, apoptosis	Breast cancer	Normal breast cell line MCF-10A, breast cancer cell line MDA-MB-435		[60]
miR-195	Cell proliferation, apoptosis, metastasis	NSCLC	A549, H129	Female athymic nude mice	[100]
miR-424	Cell proliferation angiogenesis, metastasis	Ovarian cancer	The normal human ovarian epithelial cell line HOSEpiC, human ovarian cancer cell lines (SKOV-3, HO8910, A2780), HUVECs	Immunodeficient female nude mice	[121]
miR-548c-3p	Cell proliferation, metastasis	Glioma	The human glioma T98G, U87, U251, HEK-293 cells (CRL-1573)		[68]
hsa-miR-495	Cell proliferation, metastasis	Glioma	Human glioma cell lines (A172, U87, U251, U373)		[69]
miR-150	Cell proliferation, apoptosis, cell cycle	Colorectal cancer, liver cancer, CML	K562, Meg-01, KCL-22, HL-60, KG-1; colorectal cancer cell line	Nude mice	[72, 73, 113]
miR-130a	Angiogenesis	GC	The human gastric cell line SGC7901, the human gastric mucosal epithelial cell line GES-1	Female nude mice (BALB/C-nu, 6–8 weeks)	[118]
miR-155	Angiogenesis	GC	Human SGC-7901 cells, HEK293T cells, HUVEC cell	Male nude mice (BALB/C-nu)	[120]
miR-29	Cell cycle	Breast cancer	T-47D, MDA-MB-453, MCF-7, MCF-10A cells		[61]
miR-193b-3p	Cell proliferation	T-ALL	T-ALL cell lines	T-ALL patient samples	[67]
miR-103a	Cell proliferation, metastasis	GC	MKN-45, HGC-27, MGC-803, SGC-7901, GES		[30]

CML, chronic myeloid leukemia; GC, gastric cancer; HEK, human embryonic kidney; HUVECs, human umbilical vein endothelial cells; MDA, malonaldehyde; NSCLC, non-small cell lung cancer; T-ALL, T cell acute lymphoblastic leukemia; PHFG, primary human fetal glial

MYB enhances erythropoiesis and miR-150 affects both *MYB* mRNA stability and translation efficiency [70]. *MYB*, a top predicted target of miR-150, has been fully proved. MiR-150 can induce EBV-positive BL differentiation by targeting *MYB* [71]. Moreover, miR-150 also plays a vital role in B cell development and differentiation of other hematopoietic cell lines. In chronic myeloid leukemia (CML), miR-150 can target *MYB* and inhibit the expression of a series of oncogenes, thus suppressing the proliferation of CML cells [72]. In human colorectal cancer, miR-150 also plays a tumor-suppressive role by targeting *MYB* [73]. By further focusing on the role of miRNA, new treatment strategies could be found to overcome cancers associated with elevated MYB.

### MYB interacts with lncRNAs

Abnormal expression of lncRNAs may contribute to the occurrence and development of a variety of cancers, and is partly regulated by the transcription factor MYB [74]. The expression of MYB in cancer is regulated at the level of alternative splicing, transcription, translation. Some lncRNAs can function during MYB regulation [75, 76].

### Regulation of lncRNAs by MYB

In eukaryotic cells, it is known that multifunctional MYB transcription factors regulate the expression of targeted genes by binding to specific DNA sequences [77]. The ‘-231 ~ -222’ bp region in the promoter of UCA1 is the main binding site of MYB transcription factor in hepatocellular carcinoma cells (HCC). The link between TFBs and MYB in HCC was reduced by downregulating the expression of the coactivator staphylococcal nuclease and 1-containing Tudor domain (SND1) [74]. Therefore, it is possible that regulatory effect of SND1-*MYB* complex can upregulate expression of lncRNA UCA1, thereby curbing the apoptosis levels of HCC cells [74].

### lncRNAs affecting MYB expression

**Fig. 3:**
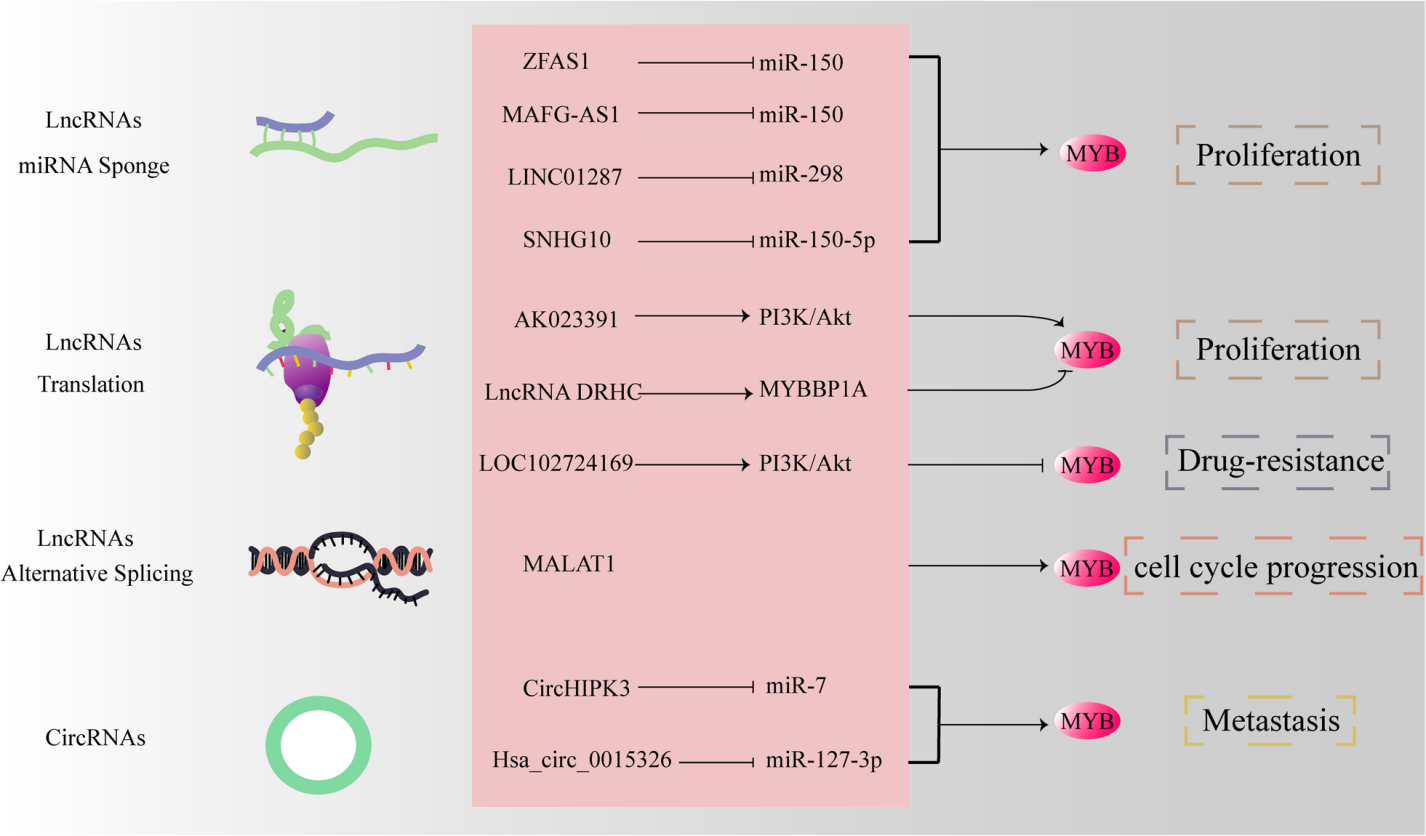
LncRNAs and circRNAs promote the expression of MYB through multiple signaling pathways. Some lncRNAs and circRNAs act as molecular sponges and bind to miRNAs, thereby upregulating the level of miRNA target genes. MALAT1 modulates the expression of cell cycle genes by regulating pre-mRNA alternative splicing

MYB deregulation has been associated with aggressive behavior in human malignancies [6]. Four lncRNAs promote the expression of *MYB* by acting as sponges of miRNAs (Table 3). The expression of LINC01287 in HCC cell lines and tissues was elevated [78]. LINC01287 plays a role as a competitive endogenous RNA (ceRNA) and negatively regulates the expression of miR-298 thus promoting the expression of *MYB*. High expression of MYB may affect cell cycle progression and promote an epithelial-mesenchymal transition (EMT) phenotype [78]. LncRNA zinc finger antisense 1 (ZFAS1) and lncRNA MAF BZIP transcription factor G antisense RNA 1 (MAFG-AS1) have been reported to be oncogenic factors in some malignancies [79, 80]. ZFAS1 and MAFG-AS1 act as molecular sponges for miR-150, resulting in downregulation of miR-150 and upregulation of *MYB* in cancers [75, 81]. High expression of lncRNA AK023391, is positively correlated with poor survival of patients with gastric cancer (GC) [82]. A study showed that cytoplasmic AK023391 is a key mediator of signal transduction in GC. lncRNA AK023391 upregulates MYB by activating the PI3K/Akt pathway, promoting GC tumorigenesis and progression [82]. Furthermore, lncRNA MALAT1 actively regulates the expression of oncogenic transcription factor MYB (Fig. 3). During the cell cycle, dynamic changes in MALAT1 levels may titrate the intracellular SR protein pool and its association with pre-mRNAs, thereby affecting the alternative splicing, stability and expression of *MYB* [76]. Interestingly, lncRNA DRHC inhibits cell proliferation, migration and invasion by binding to MYBBP1A and inhibiting *MYB*, which controls MAPK signal transduction by directly regulating the transcription of genes encoding the negative regulator SPRY2 [83]. LOC102724169 suppresses the expression of MYB in ovarian cancer with chronic stress (OCCS) by weakening PI3K/Akt signal transduction, which enhances the chemosensitivity to cisplatin and plays an antitumor role in OCCS [84]. A new study shows the carcinogenic activity of LncRNA NTT is attributed to the activation of MYB by interacting with activated complexes. The results suggest that NTT may be a new therapeutic target for the treatment of liver cancer [85].

**Table 3:** The role and mechanism of MYB-related lncRNAs in cancer

LncRNA	Molecular functions	Effects on MYB expression	Mechanism	Cellular processes	In vitro model	In vivo Model	Refs.
DRHC	Regulated transcription	Downregulated	Regulates MEK/ERK signaling	Cell proliferation, metastasis	Hh-7 and SK-Hep-1	Male BALB/C nude mice	[83]
MALAT1	Regulated transcription	Upregulated	Aggravating tumorigenesis by abnormally alternate ve splicing	Cell Cycle, cell proliferation	ELA, U2OS, HepG2, WT-MEFs WI38, WI-38-VA13, IMR-90, RKO, HCT116-WT, p532/2 cell and NIH-3T3 cell		[76]
AK023391	Regulated transcription	Upregulated	Promotes GC through activation of the PI3K/Akt pathway	Cell cycle, cell proliferation	Human GC cell lines (HGC-27, AGS, SGC-7901, BGC-823, and MGC-803) and gastric epithelial cells-1(GES-1)	Male nude mice	[82]
SNHG10	MiRNA sponge	Upregulated	Sponges miR-150-5p to decrease its suppressive effect on MYB.	Metastasis	SNU-182, Huh-7, Hep3B, SK-Hep1and SNU-38	Male athymic BALB/C nude mice	[86]
LINC01287	MiRNA sponge	Downregulated	Negatively regulates miR-298 expression	Metastasis	HepG-2, HuH7, Bel7402, Hep3B and LO2	Nude mice	[78]
LncRNA NTT	Regulated transcription	Upregulated	Activated Complex Binding	Metastasis	The HCC cell lines (Huh7 and HepG2)	BALB/C athymic nude mice	[85]
ZFAS1	MiRNA sponge	Upregulated	Regulates miR-150/MYB and miR-150/Sp1 pathways	Cell proliferation, apoptosis	Kasumi-1 cells and NB4 cells	Female nonobese diabetic/severe combined immunodeficiency (NOD/SCID) mice	[75]
MAFGAS1	MiRNA sponge	Upregulated	Regulates the miR-150-5p/MYB axis	Cell proliferation, metastasis	Breast cancer cells (MDA-MB-231, MCF-7 and MDA-MB-468) and the normal epithelial breast cell line MCF-10A	Female BALB/C nude mice	[81]
UCA1	Regulated transcription	–	SND1 regulates UCA1 expression through MYB and thus affects 5-Fu induced apoptosis of HCC cells	Apoptosis	HepG2 and SMMC-7721	Nude mice	[74]
LOC102724169	Regulated transcription	Downregulated	Inhibited MYB expression in OCCS by attenuating PI3K/AKT signaling.	Apoptosis	The human EOC cell lines SKOV3, HO8910, SKOV3/cisplatin, the normal ovarian cell line IOSE80, and the mouse EOC cell line ID8	Female nude mice and C57BL/6 mice	[84]

AML, acute myeloid leukemia; EOC, epithelial ovarian cancer; GC, gastric cancer; HCC, hepatocellular Carcinoma; MDA, malonaldehyde; OCCS, ovarian cancer with chronic stress; SND1, staphylococcal nuclease and 1-containing Tudor domain

In addition, to the more direct regulation of MYB by lncRNAs as described above, a more complex feedback loop between MYB and lncRNAs has been identified. SNHG10 eliminated the inhibitory effect of miR-150-5p on *MYB*, resulting in increased *MYB* expression [86]. Moreover, SNHG10 promotes the expression of RPL4, based on the direct interaction between RPL4 and *MYB*, which leads to an increase in *MYB* functional activity [86]. Reciprocally, overexpression and overactivation of *MYB* enhance the expression of SNHG10 and SCARNA13 by binding to the promoter region of SNHG10 [86]. Collectively, SNHG10 regulates the expression of SCARNA13 through the miR-150-5p/RPL4-MYB positive feedback loop to facilitate the development and progression of HCC [86].

### MYB interacts with circRNAs

CircRNAs are a class of ncRNA molecules without a 5'-end cap and a 3′-end poly (A) tail [87], and they are formed by covalent bonds with a circular structure [38]. They are widely diverse endogenous RNA molecules that regulate gene expression in eukaryotic cells [88]. Functionally, circRNAs act as transcriptional regulators to control the expression of host genes [89, 90]. CircRNAs are closely associated with human diseases, especially cancers, and may be better biomarkers due to their abundance and stability [91–94]. Recent studies have shown that circRNAs are rich in miRNA binding sites and act as miRNA sponges in cells, thereby relieving the inhibitory effect of miRNAs on their target genes and thus increasing their expression [95] (Fig. 3). Interestingly, the differential expression circRNA back-spliced from *MYB* gene can act as a sponge of miRNA and play a vital role in diseases [96]. Among patients with colorectal cancer, the overexpression of *MYB* promotes the transcription of circHIPK3 and circHIPK3 has oncogenic functions by sponging miR-7 [97]. Moreover, hsa_circ_0015326 sponges miR-127-3p to regulate MYB signaling, which is closely related to the occurrence and development of ovarian cancer [98]. In summary, hsa_circ_0015326 positively regulates MYB signaling and acts as a tumor-promoting factor; thus, its downregulation could be a potential therapeutic approach [98].

### Role of non-coding RNAs and MYB in cancers

Multiple ncRNAs play a crucial role in cell processes and tumorigenesis. The interaction between ncRNAs and MYB is involved in tumor cell proliferation, apoptosis, angiogenesis, metastasis, senescence, and drug resistance (Fig. 1).

#### Proliferation

**Fig. 4:**
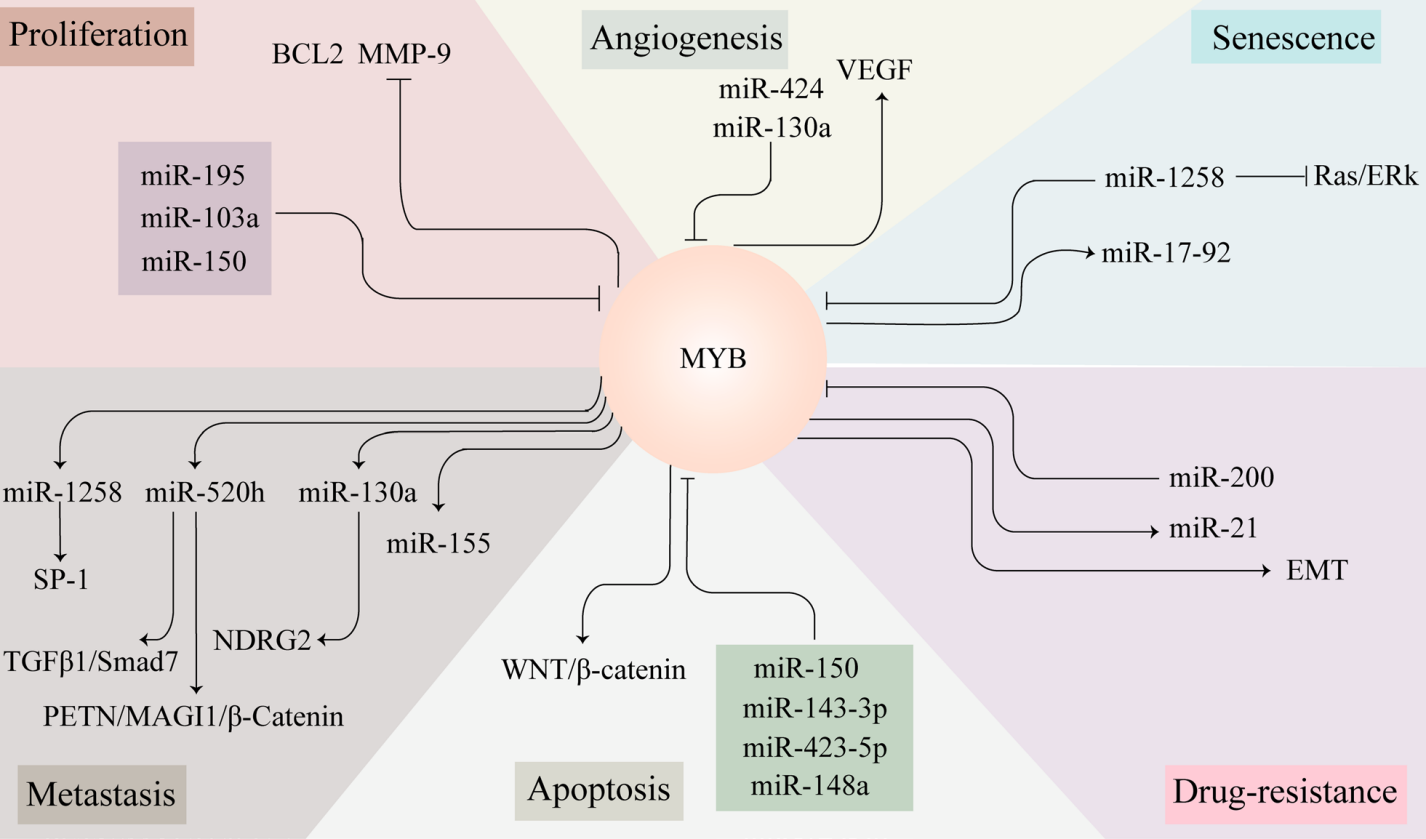
The interaction between miRNAs and MYB is involved in tumor cell proliferation, apoptosis, angiogenesis, metastasis, senescence and drug resistance. LncRNAs promote the expression of MYB through multiple signaling pathways. **A** Some lncRNAs act as molecular sponges and bind to miRNAs, thereby upregulating the level of miRNA target genes. **B** LncRNAAK023391 plays its role by activating signaling pathways. **C** MALAT1 promotes cell proliferation by regulating pre-mRNA alternative splicing

The unlimited proliferation of cancer cells contributes to their malignant phenotype and affects the prognosis of patients. Increasing evidence suggests that MYB has survival-promoting functions. Some miRNAs impede the function of MYB, thereby inhibiting cancer cell proliferation (Fig. 4). Matrix metalloproteinases (MMPs) belong to the protease family and have been shown to play a key role in tissue remodeling and supporting cancer development [99]. In non-small-cell lung cancer (NSCLC), miR-195 directly targets *MYB* 3'UTR and negatively regulates its expression, thereby regulating the proliferation and metastasis of tumor cells [100]. In addition, *MYB* gene deletion can inhibit the expression of BCL2 and MMP-9 [101]. Meanwhile, MMP-1 and MMP-9 were downregulated along with BCL2 and CCNE1 in A549 and H1299 cells transfected with miR-195. In general, this suggests that miR-195 at least partially reduces the expression of BCL2 and MMP-9 through MYB [100]. Recent studies indicate that MYB plays an essential role in the development and progression of GC [102]. MYB was identified as the functional downstream target of miR-103a, and its ectopic expression partially reversed the inhibition of cell proliferation and invasion. Therefore, miR-103a regulates the development of tumors by regulating *MYB* [30, 103]. Many studies have confirmed that miR-150 regulates MYB and affects the proliferation of various types of tumor cells [71, 104, 105]. Mitogen-activated protein kinase (MAPK) signal transduction is a highly conserved signaling pathway involved in a variety of biological events, including metabolic reprogramming, cell proliferation, survival, and differentiation. Mutations in key molecules involved in MAPK/ERK signaling and maladjustment of this pathway are common events in many human malignancies [106]. MAPKs in mammals include JNK, p38 and ERK. MAPK/ERK signaling pathway plays a key role in tumorigenesis and development by promoting cell proliferation and metastasis. In hepatocellular carcinoma, lncRNA DRHC interacts with MYBBP1A and regulates the proliferation of hepatoma cells by regulating MEK/ERK signaling through *MYB*. However, the exact mechanism of lncRNA DRHC/MYBBP1a/ MYB is not clear [83].

#### Apoptosis

Apoptosis plays a vital role in maintaining tissue homeostasis, and imbalance of the apoptosis pathway is considered a critical step in tumorigenesis [107]. Many studies have found that the interaction between MYB and ncRNAs can regulate tumor cell apoptosis (Fig. 4). MYB is involved in cancer progression and has become an important target of various miRNAs, such as miR-423-5p and miR-143-3p [60, 108]. In colorectal cancer, knocking down *MYB* can promote the expression of miR-148a, and knockout of *MYB* can also partially induce apoptosis of cancer cell lines [46]. More interestingly, the expression of miR-30b-5p is significantly downregulated in medulloblastoma (MB) cells. miR-30b-5p inhibits MB progression by targeting the expression of MYB [109]. A study showed that melatonin inhibits the expression of miR-155, thus inhibiting the proliferation, migration and invasion of glioma cells. It has been suggested that melatonin may be a therapeutic strategy for MYB-miRNA-induced glioma [110]. A negative correlation between MYB and miR-143-3p expression was found in breast cancer tissues and cells. More importantly, MYB is involved in regulating the proliferation and apoptosis of breast cancer cells [60]. Antisense miR-148a inhibitors can restore down-regulated MYB-induced apoptosis [46]. To sum up, MYB seems to be the key regulator of miR-148a promoting apoptosis in colorectal cancer cells [111]. In the case of glioblastoma, some studies have illustrated that miR-148a acts as a negative risk factor. Upregulated miR-148a could accelerate malignant process and is negatively correlated with the survival rate [111]. Some studies have proved that isomorphic diffuse gliomas have MYB/MYBL1 changes, thus MYB plays an important role in the development of glioblastoma [112]. We speculate that there may be a close relationship between miR-148a and MYB in gliomas. A negative correlation between MYB and miR-143-3p expression was found in breast cancer tissues and cells. More importantly, MYB is involved in regulating the proliferation and apoptosis of breast cancer cells [60]. The overexpression of miR-150 increases the apoptosis of CD133^+^ hepatoma cells. MiR-150 inhibits the expression of *MYB*, leading to changes in several key proteins related to the cell cycle and cell survival, including cyclin D1 and BCL-2 [113]. SND1 are evolutionarily conserved proteins that exist in eukaryotic cells from protozoa to mammals. SND1 is becoming increasingly important because it is overexpressed in invasive cancer cells and a variety of primary tumors. Currently, it is considered to be a sign of malignancy [114]. Studies have shown that MYB protein, which binds SND1 protein, may act as a transcription factor of lncRNA UCA1 in vitro. In addition, SND1 may upregulate the expression of lncRNA UCA1 by acting as a coactivator of MYB, thus affecting the apoptosis of HCCs [74].

#### Angiogenesis

Angiogenesis plays an important role in the development and metastasis of tumors, and inhibition of this process will prevent the development and diffusion of tumor tissues [115, 116]. Given the critical position of angiogenesis in tumor formation and development, it is of great significance to find new anti-vascular targets. A great deal of evidence indicates that the interaction between ncRNAs and MYB can affect angiogenesis in numerous tumors. As a transcription factor, MYB is related to various intracellular biological behaviors and is closely related to the process of angiogenesis (Fig. 4) [117]. *MYB* is the direct target of miR-130a. Cancer-derived exosomes carry miR-130a from GC cells to vascular cells by targeting *MYB* to promote angiogenesis and tumor growth [118]. Vascular endothelial growth factor A (VEGF) is the primary mediator of angiogenesis, and VEGF directly contributes to targeting tumor cell growth and metastasis [119]. In addition, other studies have found a negative correlation between the expression of miR-155 and *MYB* in gastric cancer [120]. More importantly, experiments have shown that MSCs can transport miR-424 to ovarian cancer cells to target MYB to further inhibit the expression of VEGF and the proliferation, migration and tube formation of endothelial cells, to block angiogenesis [121].

#### Metastasis

Metastasis is known to be the leading cause of cancer-related deaths and is a considerable challenge in cancer treatment [122, 123]. In addition to cooperating with protein-coding promoters, MYB also enhances the activity of ncRNA promoters to facilitate cancer initiation and metastasis [48]. MAGI1 is a member of a protein family, that plays an important role in coupling the extracellular environment with intracellular signaling pathways and the cytoskeleton at synapses and tight junctions. One piece of evidence confirmed the key role of MAGI1 in regulating cell–cell contact, which is always destroyed in tumor progression and related to invasiveness and metastasis [124]. In recent years, studies have shown that MAGI1 can be directly targeted by miR-520-h in renal cell carcinoma (RCC) cells [48]. At the same time, MYB promotes the transcriptional activity of miR-520-h by binding to the RCC promoter to regulate MAGI1 expression, and the overexpression or knockout of MAGI1 regulates PETN/MAGI1/β-Catenin and significantly affects the invasion and migration of human renal cell carcinoma cells [48]. In epithelial ovarian cancer (EOC), miR-520-h promotes EOC progression by activating TGF-β1/Smad7 signal transduction pathway. Overexpression of Smad7 attenuated the oncogenic effect of miR-520-h [125]. More importantly, in EOC, TGF-β1 increases the expression of miR-520-h by upregulating its upstream transcription factor MYB [48, 125]. NDRG2 is a critical anticancer gene in salivary adenoid cystic carcinoma (SACC), which contributes to inhibiting cell proliferation and metastasis of SACC. A study has confirmed that MYB is a crucial driver by which SACC overexpresses miR-130a, thereby inducing downregulation of NDRG2 [47]. In addition, miR-1258 has been found to have an inhibitory effect on a variety of cancers. In oral squamous cell carcinoma (OSCC), MYB inhibits miR-1258 by directly binding to the miR-1258 promoter [49]. Dysregulated miR-1258 promotes the expression of SP-1 protein, which contributes to the development of OSCC [49]. Importantly, there is evidence that SP-1 plays a role in cancer progression, invasion and metastasis. SP-1 can promote cell proliferation by accelerating the cell cycle from G1 to S phase [126, 127]. In human LSCC, YB-1 transcription factors promote the invasion and migration of cancer cells through MYB-induced miR-155 expression [54]. In addition, the abnormal expression of YB-1/MYB/miR-155 promotes the progression of laryngeal carcinoma and is related to poor prognosis [54]. Therefore, YB-1 can be considered as a potential prognostic and therapeutic target for patients with laryngeal cancer. Moreover, MYB upregulates circRNAs at the transcriptional level, such as circHIPK3, which acts as a novel oncogenic circRNA by sponging miR-7. MYB inhibits the expression of circHIPK3, and the metastasis of cancer cells can be controlled [97]. In summary, the above studies show that it is urgent to deeply understand the complex relationship between MYB and ncRNAs. This is very important for the metastasis of cancer cells in vivo and an important strategy to control the development of cancer cells.

#### Senescence

Growing evidence suggests that MYB is a potential candidate for the regulation of senescence, and inhibition of MYB expression plays an essential role in the growth arrest of senescence (Fig. 4) [128]. MYB inhibits the expression of miR-1258. When MYB is suppressed, the overexpression of miR-1258 inhibits the expression of GRB2 and then inactivates the carcinogenic pathway of Ras/ERK, which then induces senescence and apoptosis of tumor cells [49, 129]. Moreover, miR-17-92 promotes tumorigenesis by antagonizing oncogene-induced senescence [130]. MYB significantly adjust the expression of miR-17-92 targets such as p21, a key effector of senescence. When *MYB* is silenced, the survival of cells can be suppressed [131].

#### Drug-resistance

Drug resistance is another major clinical challenge in cancer treatment. In breast cancer, MYB induces EMT and significantly increases tamoxifen resistance. Given the ability of miR-200 to control gene expression, it has emerged as an important role in response to anticancer therapies, particularly in the development of drug resistance (Fig. 4). Experiments have shown that miR-200 inhibits the expression of MYB, reversing the drug resistance of cancer cells to tamoxifen. This might be the result of miR-200-MYB regulating EMT [58]. Interestingly, MYB can activate the expression of miR-200 through a transcriptional, binding-dependent mechanism. It may also be related to drug resistance [59]. Moreover, in ovarian cancer, high MYB expression can cause tumor cells to resist cisplatin. Silencing *MYB* reduced the miR-21 level and EMT, which reverses cisplatin resistance [132].

### Potential clinical application of MYB and noncoding RNAs in cancer

Due to the increasing knowledge about the biology and function of MYB and ncRNAs and the emergence of new treatment opportunities, some drugs can make use of a variety of mechanisms, directly and indirectly, and affect the relationship between MYB and ncRNAs in different ways, to inhibit the growth and metastasis of tumor cells [110, 133].

#### Targeting MYB

In recent years, several approaches have been attempted to inhibit abnormal MYB expression in cancer cells. The initial attempt was to use RNA interference (RNAi) to inhibit *MYB*. In a mouse model of MLL-AF9 leukemia, *MYB* specific shRNA effectively silenced MYB and showed that its inhibition could eradicate invasive leukemia in vivo without affecting normal myelopoiesis [134]. Another study found that a *MYB* DNA vaccine in combination with an anti-PD-1 antibody or low dose cyclophosphamide effectively extended survival of colorectal cancer (CRC) bearing mice [135]. Moreover, important coactivators and degradation regulators of MYB have been investigated as therapeutic targets. Recently, mebendazole has been shown to effectively inhibit in vivo progression of AML by interfering with the heat shock protein 70 (HSP70) chaperone system and inducing MYB degradation by proteasome [136]. Many studies have shown that melatonin has significant apoptotic, angiogenesis, antitumor and antiproliferation effects on many kinds of tumor cells [137]. In gliomas, melatonin may affect the expression of MYB to inhibit miR-155, thus inhibiting the proliferation, migration and invasion of glioma cells. Therefore, correlation between melatonin and MYB/miR-155 may provide a new strategy for the treatment of human gliomas [110].

#### Targeting ncRNAs

In fact, in addition to our above treatment strategies for MYB, ncRNAs mentioned in this review can also be used as a target for cancer treatment. For example, antisense miR-155 molecule cobomarsen (MRG-106) uses LNA-modified antisense oligodeoxynucleotides to inhibit miR-155 in the treatment of T-cell lymphoma, which means that oligonucleotides composed of LNA may be a valuable ncRNAs detection tool in cancer diagnosis and prognosis. A Phase I clinical trial of cobomarsen was launched in 2016 [138]. A study showed that targeted delivery of miR-34a mimics using lipid emulsions significantly inhibited cancer progression in a xenograft mouse model of colon cancer [139]. Therefore, MRX34, a miR-34a liposome injection, entered a phase I clinical trial in 2013. Although the experiment was ultimately terminated, the development of MRX34 showed feasibility [140]. Moreover, ginkgetin is a natural nontoxic biflavone, that has been proven to have anti-cancer, anti-inflammatory, anti-microbial, anti-adipogenesis and neuroprotective activities [141]. Ginkgetin can combat cancer progression by blocking cell cycle, inducing apoptosis, stimulating autophagy and targeting many dysfunctional signaling pathways [141]. In colon cancer, ginkgo flavonoids regulate the expression of miR-34a to regulate the expression of MYB, which can induce G2 phase arrest and apoptosis of colorectal cancer cells [133]. In summary, ncRNAs targeting MYB show great hope in preliminary studies. A better understanding of MYB and its regulation of ncRNA activity and expression to select effective inhibitors should help to improve the survival rate of patients with MYB-related tumors.

## Conclusion

There are several mechanisms to activate MYB in human cancer. In general, these mechanisms will lead to higher levels or more transcriptionally active MYB, and to persistent expression. The ability of MYB to block differentiation seems to be responsible for this sustained expression. Moreover, tumor cells are “addicted” to the higher level of MYB. Therefore, MYB transcription factor is a suitable target for tumor therapy. However, the lack of effective MYB-specific inhibitors has been a significant problem in clinical studies. The development of MYB-targeted regulation will help improve survival in patients with MYB-related tumors. MYB is also regulated by multilayered network of ncRNAs with multiple ncRNAs. NcRNAs act as modulators of MYB, and they regulate MYB at the transcription, translation, protein stability, and functional levels through various mechanisms. Reciprocally, ncRNAs can also act as effectors of MYB and even form feedback loops with MYB. Furthermore, given the known tissue specificity of ncRNAs expression, the involvement of ncRNAs as MYB cofactors may become a new potential target to control MYB expression. This method of identifying the addiction of oncogenes to cancer and aiming to control the regulatory mechanisms driving oncogene expression may be a new approach of anticancer drugs. Moreover, the ncRNA-MYB coregulatory network brings a systematic and enlightening point of view for the regulation of gene expression in cancer prognosis. Evidence suggests that treatment for miRNA and TFs has a broader effect compared with treatment for a single gene. The combination of miRNA mimics and inhibitors targeting the same oncogene can produce synergy, prolong the effective treatment window and may bring better therapeutic effects. Importantly, synergy can produce similar or better efficacy at lower inhibitor dosages, help to improve the specificity of combination therapy and reduce the toxicity and side effects at higher doses [142]. Therefore, further research is needed to develop effective therapeutic interventions aimed at inhibiting MYB-related oncogene signaling in tumors while minimizing the risk to patients.

## Data Availability

Not applicable.

## Abbreviations

V-MYB: Virus MYB
AMV: Avian myeloblastosis virus
DBD: DNA-binging domain
NRD: Negative regulatory domain
TAD: Transactivation domain
c-MYB: Cellular MYB
HSC: Hematopoietic stem cell
MBS: MYB binding site
ncRNAs: Noncoding RNA
LncRNA: Long-non-coding RNA
miRNA: MicroRNA
circRNA: Cyclic RNA
mRNA: Messenger RNA
B-CLL: B-lymphocytic leukemia
TF: Transcription factor
UTR: Untranslated region
TFBs: Transcription factor binding sites
MAGI1: Membrane-associated guanylate kinase and reverse repeat member 1
AML: Acute myeloid leukemia
YB-1: Y-box binding protein 1
LSCC: Laryngeal squamous cell carcinoma
CML: Chronic myeloid leukemia
HCC: Hepatocellular carcinoma cells
SND1: Staphylococcal nuclease and 1-containing Tudor domain
ceRNA: Competitive endogenous RNA
EMT: Epithelial-mesenchymal transition
ZFAS1: Zinc finger antisense 1
MAFG-AS1: MAF BZIP transcription factor G antisense RNA 1
GC: Gastric cancer
OCCS: Ovarian cancer with chronic stress
MMPs: Matrix metalloproteinases
NSCLC: Non-small-cell lung cancer
MAPK: Mitogen-activated protein kinase
MB: Medulloblastoma
VEGF: Vascular endothelial growth factor A
RCC: Renal cell carcinoma
EOC: Epithelial ovarian cancer
SACC: Salivary adenoid cystic carcinoma
OSCC: Oral squamous cell carcinoma
RNAi: RNA interference
CRC: Colorectal cancer
HSP70: Heat shock protein 70
